# Small Hepatoid Variant of Solid Pseudopapillary Neoplasm of the Pancreas: A Unique Case With Long‐term Follow‐up

**DOI:** 10.1002/deo2.70310

**Published:** 2026-03-03

**Authors:** Hisakazu Matsumoto, Kosuke Minaga, Shotaro Ueno, Asuka Sone, Eiki Chikugo, Kohei Tonomura, Shogo Nakano, Yoshito Uenoyama, Kazuo Ono, Yukitaka Yamashita

**Affiliations:** ^1^ Department of Gastroenterology Japanese Red Cross Wakayama Medical Center Wakayama Japan; ^2^ Department of Gastroenterology and Hepatology Kindai University Faculty of Medicine Osaka Japan; ^3^ Department of Diagnostic Pathology Japanese Red Cross Wakayama Medical Center Wakayama Japan

**Keywords:** hepatoid tumor, pancreatic incidentaloma, pancreatic tumor, solid pseudopapillary neoplasm, solid pseudopapillary tumor

## Abstract

Hepatoid tumors are rare neoplasms that arise outside the liver but exhibit morphological and immunophenotypic features resembling hepatocellular carcinoma. Although hepatoid differentiation sometimes occurs in pancreatic ductal adenocarcinoma, its occurrence in solid pseudopapillary neoplasm (SPN) is exceedingly rare. Consequently, the clinicopathological characteristics and natural history of this variant remain poorly understood. We report a case of a 37‐year‐old asymptomatic male with a 6 mm solid pancreatic body lesion that was incidentally detected during routine abdominal ultrasonography. Multimodal imaging showed a well‐circumscribed solid mass showing early‐phase hyperenhancement relative to the surrounding pancreatic parenchyma. Tissue samples obtained via endoscopic ultrasonography contained polygonal epithelioid cells with abundant eosinophilic cytoplasm and a hepatoid appearance. In immunohistochemistry, HepPar‐1 and CD10 were diffusely positive, chromogranin A and synaptophysin were absent, and β‐catenin accumulated within the nucleus, all supporting a diagnosis of SPN with hepatoid differentiation. Although surgical resection was recommended, the patient declined and was subsequently managed with active surveillance. After 5 years of follow‐up, the lesion remained morphologically stable without clinical progression. To our knowledge, this case report is the first to describe the long‐term natural course of a pancreatic hepatoid SPN managed nonoperatively. The patient's maintained stability supports the indolent biological behavior of this rare variant. Thus, conservative management with close surveillance may be feasible for carefully selected patients with small, asymptomatic tumors. However, additional cases are needed to clarify optimal management strategies.

## 1 Introduction

The widespread use and improved resolution of advanced imaging modalities have led to the incidental detection of pancreatic lesions in otherwise asymptomatic individuals [1]. These pancreatic incidentalomas, encompassing cystic and solid abnormalities, represent a broad clinicopathological spectrum ranging from benign to malignant lesions, thereby posing substantial diagnostic and management challenges. Small solid pancreatic lesions, particularly those ≤10 mm, present a distinctive diagnostic dilemma: imaging alone frequently fails to provide reliable etiologic characterization, yet accurate diagnosis is essential for determining appropriate management, including surgical resection, local ablation, or active surveillance.

Endoscopic ultrasonography (EUS) provides high‐resolution assessment of the pancreatic parenchyma, and subsequent EUS‐guided tissue acquisition (EUS‐TA) enables histopathological confirmation, making EUS a central diagnostic modality for small solid pancreatic tumors. Nonetheless, lesion size remains a major determinant of diagnostic performance. A recent meta‐analysis involving 6883 cases demonstrated that EUS‐TA has significantly higher sensitivity and accuracy for lesions >10 mm than for subcentimeter lesions, underscoring the difficulty of diagnosing very small tumors [2].

This report describes a rare case of an incidentally detected small solid pancreatic lesion in a young male, diagnosed by EUS‐TA as a hepatoid variant of solid pseudopapillary neoplasm (SPN) and managed with active surveillance for 5 years.1

## 2 Case Report

A 37‐year‐old asymptomatic man with a small pancreatic mass that was incidentally detected by abdominal ultrasonography during a routine health examination was referred to our hospital for evaluation. He had no significant medical history. Results of different laboratory tests, including alpha fetoprotein, carcinoembryonic antigen, and carbohydrate antigen 19‐9, were all normal.

**FIGURE 1:**
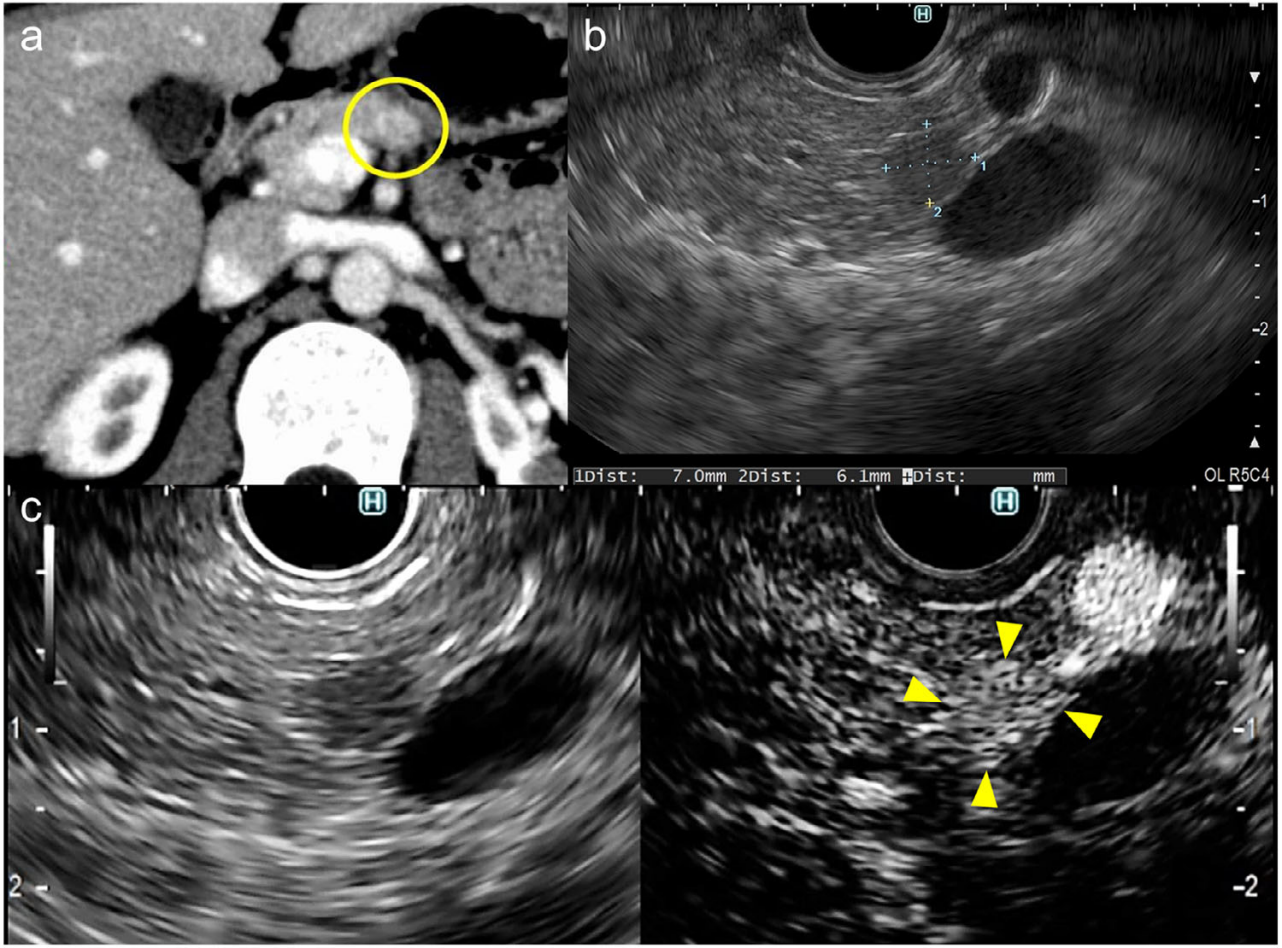
Imaging findings of the small pancreatic body lesion. (a) Contrast‐enhanced computed tomography demonstrating an early‐enhancing nodule in the pancreatic body (circled area) during the arterial phase. (b) Endoscopic ultrasonography revealing a 7 mm well‐defined, round‐to‐oval, hypoechoic mass. (c) Contrast‐enhanced endoscopic ultrasonography showing early‐phase hyperenhancement of the mass relative to the surrounding pancreatic parenchyma (arrowheads); the left panel shows the monitor image, and the right panel shows the contrast‐enhanced image.

**FIGURE 2:**
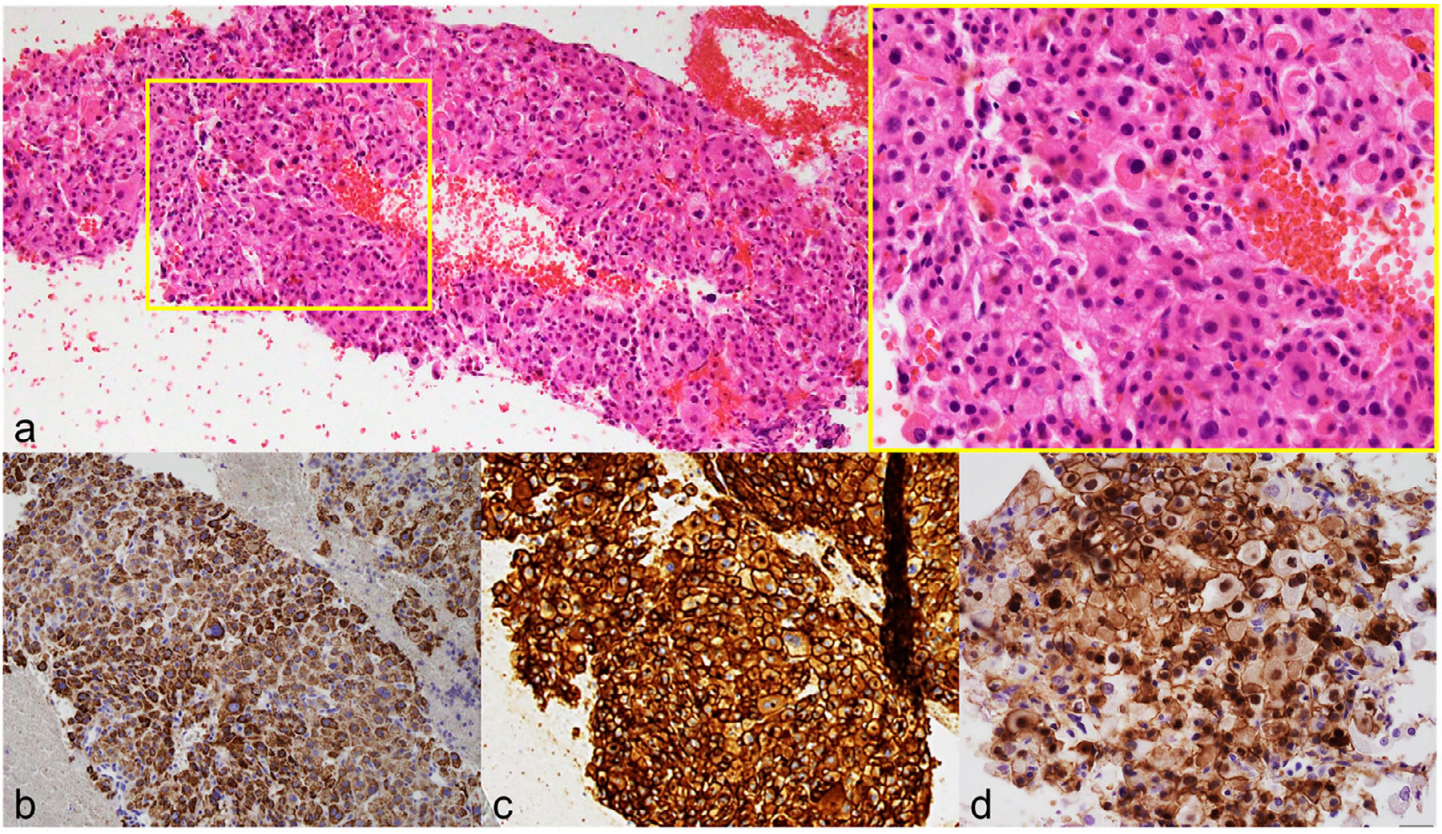
Histopathological and immunohistochemical features of the pancreatic body lesion. (a) Hematoxylin and eosin staining showing diffuse epithelioid proliferation comprising polygonal cells with abundant eosinophilic cytoplasm and a hepatoid appearance. (b–d) Immunohistochemical staining demonstrating diffuse positivity for HepPar‐1 (b) and CD10 (c), and nuclear β‐catenin accumulation (d).

Abdominal ultrasonography showed a 6 mm low‐echoic mass in the pancreatic body. On contrast‐enhanced computed tomography (CT), an early‐enhancing nodule emerged in the pancreatic body during the arterial phase (Figure 1a). Meanwhile, no abnormal tracer uptake within the lesion was detected on ^111^In‐pentetreotide single‐photon emission computed tomography. Subsequently, EUS was performed using a curvilinear‐array echoendoscope (GF‐UCT260; Olympus Medical Systems, Tokyo, Japan), revealing a well‐defined, round‐to‐oval, hypoechoic mass in the pancreatic body (Figure 1b). Contrast‐enhanced EUS demonstrated early‑phase hyperenhancement of the pancreatic mass relative to the surrounding parenchyma (Figure 1c). EUS‐TA was subsequently performed using a 22‐gauge needle (EZ Shot 3 Plus; Olympus Medical Systems) with a slow‐pull technique. Rapid on‐site evaluation with quick Papanicolaou staining was performed. Adequate diagnostic material consisting of atypical cells with nuclear enlargement was confirmed, and therefore, the procedure was completed after two passes. Tissue specimens stained with hematoxylin and eosin revealed diffuse epithelioid proliferation involving polygonal cells with abundant eosinophilic cytoplasm (Figure 2a). These cells exhibited a hepatoid pattern, closely resembling hepatocellular carcinoma (HCC). Prominent nucleoli, anisokaryosis, and focal necrosis were also noted.

Immunohistochemically, HepPar‐1 (Figure 2b) and CD10 (Figure 2c) were diffusely positive, supporting hepatoid differentiation. The tumor cells were also positive for AE1/AE3 and CD56 but negative for CK7, CK20, CD34, IMP3, and CEA. No neuroendocrine markers, including chromogranin A and synaptophysin, were seen. Furthermore, β‐catenin clearly accumulated in the nucleus—a characteristic feature of SPN (Figure 2d). The Ki‐67 labeling index was below 1%. The histopathological and immunophenotypic findings supported the diagnosis of pancreatic SPN with hepatoid differentiation. Although metastatic HCC could not be completely ruled out according to histology alone, it was highly unlikely because of no prior history of HCC and no radiological evidence of hepatic lesions at presentation.

Given the malignant potential of pancreatic SPN, surgical resection was recommended. However, the patient refused, and a strategy of careful observation was subsequently adopted. Contrast‐enhanced CT and EUS have since been performed alternately every 6 months. After 5 years of follow‐up, the patient remains asymptomatic, and the tumor has not changed morphologically.

## 3 Discussion

Hepatoid tumors are exceptionally rare neoplasms that emerge outside the liver but display histological and immunohistochemical features resembling HCC [3]. The World Health Organization classifies hepatoid tumors of the pancreas as potential variants of pancreatic ductal adenocarcinoma (PDAC). Importantly, several clinicopathological reviews have indicated that PDACs with hepatoid differentiation frequently exhibit aggressive biological behavior, characterized by prominent vascular invasion, early liver and lymph node metastasis, and poor prognosis compared with conventional PDAC [3, 4].

In 2022, Mattiolo et al. reported two cases of pancreatic SPN with hepatoid differentiation and proposed this phenotype as a new variant within the SPN spectrum [5]. SPN is a low‐grade malignant tumor that accounts for approximately 1%–2% of all pancreatic neoplasms and predominantly affects young females [6]. It is genetically distinctive, typically harboring activating *CTNNB1* mutations that lead to nuclear and cytoplasmic β‐catenin accumulation [3, 6]. Regarding the cases reported by Mattiolo et al., the tumors emerged in the pancreatic head and tail in males aged 56 and 53 years. Both lesions demonstrated a pure hepatoid morphology with strong HepPar‐1 and CD10 expression. The absence of chromogranin A and synaptophysin, which are key markers of neuroendocrine neoplasms, and the nuclear positivity for β‐catenin and LEF‐1 supported the diagnosis of SPN. Additionally, molecular analysis identified somatic *CTNNB1* mutations, consistent with the hepatoid SPN as a distinct entity [5].

Clinically, the two reported patients with the hepatoid SPN remained disease‐free for 12.3 years and 10 months, respectively, indicating indolent behavior despite their relatively large tumor sizes (5 and 3.7 cm). Thus, although hepatoid differentiation in PDAC has been associated with aggressive behavior [3, 4], the available evidence does not indicate that hepatoid differentiation itself confers a higher malignant potential in SPN. Instead, the clinical significance of hepatoid morphology appears to be determined by the underlying tumor entity rather than by hepatoid differentiation alone.

Before Mattiolo et al.’s report [5], Akimoto et al. reported a 59‐year‐old male with a 4.5 cm pancreatic body tumor diagnosed as pancreatic hepatoid carcinoma mimicking SPN [7]. The immunophenotypic profile, which included HepPar‐1 and CD10 positivity, absent neuroendocrine markers, and nuclear β‐catenin accumulation, strongly supports the classification of this case as a hepatoid SPN. Contrast‐enhanced EUS also revealed the lesion's hypervascularity [7], a finding consistent with the present case. Regarding imaging findings, hepatoid carcinoma of the pancreas has been reported to exhibit HCC‐like enhancement patterns, including early arterial‑phase enhancement on contrast‑enhanced CT, which is uncommon in conventional PDAC [8]. Similarly, although typical SPNs are generally considered hypovascular, both the present case and the case reported by Akimoto et al. demonstrated relative hypervascularity on contrast‑enhanced EUS. These observations raise the possibility that hepatoid differentiation may influence tumor vascularity.

Complete surgical resection is the standard treatment for pancreatic SPN because of its malignant potential, yielding an excellent 5‐year survival rate exceeding 95% [6]. Although SPN is classified as a low‑grade malignant tumor, its biological behavior is heterogeneous. Uchimi et al. reported that non‑cystic SPNs were significantly smaller and less frequently symptomatic than classic cystic tumors, with a low incidence of overt malignancy [9]. Notably, their series included a young patient with biopsy‑proven non‑cystic SPN who was managed without surgical resection and showed no progression during follow‑up, providing rare insight into the natural history of untreated SPN [9]. Such biological heterogeneity and the generally favorable behavior observed in selected cases have prompted interest in less invasive management strategies for small SPNs. Recently, EUS‐guided radiofrequency ablation has emerged as a minimally invasive, tissue‐preserving treatment option for small SPNs [10]; however, evidence regarding its indications and long‐term outcomes remains limited.

To our knowledge, this report is the first to describe the long‐term natural course of a pancreatic hepatoid SPN managed without surgical resection. The sustained clinical and radiological stability observed over 5 years demonstrates the indolent biological behavior of this exceptionally rare variant. Therefore, in carefully selected patients with small and asymptomatic tumors, a conservative management strategy with close surveillance may be a reasonable option. Nevertheless, surgical resection should be reconsidered if radiological progression is observed, including tumor growth, the development of cystic changes, or the emergence of invasive features. Further accumulation of cases is needed to establish appropriate management strategies.

## Author Contributions

**Hisakazu Matsumoto**: patient care, data acquisition, interpretation of the data, and drafting of the manuscript. **Kosuke Minaga**: drafting of the manuscript, literature review, and critical revision of the manuscript for important intellectual content. **Kazuo Ono**: pathological evaluation and interpretation of histopathological findings. **Shotaro Ueno**, **Asuka Sone**, **Eiki Chikugo**, **Kohei Tonomura**, **Shogo Nakano**, and **Yoshito Uenoyama**: interpretation of the data and manuscript review. **Yukitaka Yamashita**: supervision of the study.

## Funding

The author has received no specific funding for this work.

## Consent

Informed consent was obtained from the patient for the medical treatment and procedures described. In publication, all accompanying images have been anonymized to ensure that the individual cannot be identified.

## Conflicts of Interest

The authors declare no conflicts of interest.

## Supporting information




**Supporting file 1**: deo270310‐sup‐0001‐SuppMat.pdf
